# Zinc Influences the Efficacy of Betulinic Acid Treatment and Radiotherapy in Breast Cancer Cells

**DOI:** 10.3390/antiox13111299

**Published:** 2024-10-25

**Authors:** Antje Güttler, Elisa Darnstaedt, Danny Knobloch-Sperlich, Marina Petrenko, Jacqueline Kessler, Ivo Grosse, Dirk Vordermark, Matthias Bache

**Affiliations:** 1 Department of Radiotherapy, Martin Luther University Halle-Wittenberg, Ernst-Grube-Str. 40, 06114 Halle, Germany; elisa_darnstaedt@aol.com (E.D.); danny.knobloch-sperlich@uk-halle.de (D.K.-S.); marina.petrenko@uk-halle.de (M.P.); jacqueline.kessler@uk-halle.de (J.K.); dirk.vordermark@uk-halle.de (D.V.); matthias.bache@uk-halle.de (M.B.); 2 Institute of Computer Science, Martin Luther University Halle-Wittenberg, Von-Seckendorff-Platz 1, 06120 Halle, Germany; grosse@informatik.uni-halle.de; 3 German Centre for Integrative Biodiversity Research (iDiv) Halle-Jena-Leipzig, Puschstrasse 4, 04103 Leipzig, Germany

**Keywords:** breast cancer, betulinic acid, zinc, ROS, radiation, radiosensitivity

## Abstract

The trace element zinc influences a number of biological reactions, including cell growth, apoptosis, and DNA damage, which affect tumor therapy. The natural compound betulinic acid (BA) and its derivatives are known for their antiviral, antibacterial, and antitumor effects. Previous studies show that BA and 3-acetyl-28-sulfamoyloxybetulin (CAI3) have high cytotoxicity and induce radiosensitization in breast cancer cells. This study investigates the effects of zinc supplementation on treatment with BA or CAI3 and radiotherapy of breast cancer cell lines MDA-MB-231 and HS578T. Expression analysis shows that BA and CAI3 lead to altered expression of genes involved in zinc metabolism. Zinc supplementation affects cell survival and cell death alone and in combination with BA or CAI3 in both breast cancer cell lines. In MDA-MB-231 cells, zinc excess protects against ROS formation by BA or CAI3 and exhibits radioprotective effects compared to the single agent treatment. In contrast, in HS578T cells, zinc induces ROS formation but does not affect radiosensitivity. The variable effects of zinc on radiosensitivity highlight the importance of individualized treatment approaches. Although zinc has cytotoxic, pro-apoptotic, and anti-clonogenic effects, it seems worthwhile to consider its radioprotective properties when making treatment decisions in the case of adjuvant radiotherapy of breast cancer.

## 1. Introduction

In the ‘Global Cancer Statistics 2020’, breast cancer is listed as the most commonly diagnosed cancer worldwide, and it is the second leading cause of cancer-related deaths in women [1]. There are various factors that influence breast cancer risk, including gender, age, genetic mutations, and family history [2,3,4]. Depending on tumor stage and subtype, different treatment approaches are considered, with the three main pillars being surgery, radiation, and chemotherapy. However, radiotherapy and chemotherapy often come with significant side effects such as a weakened immune system, hair loss, and nausea, leading to an ongoing search for new therapeutic options. Targeted therapies are gaining importance, using substances that specifically target certain characteristics of tumor cells.

Pentacyclic triterpenes, such as betulin or betulinic acid (BA), have long been used in various therapies. BA exhibits a wide range of activities, including antiviral, antibacterial, and antitumor effects [5,6]. Its cytotoxic activity is selective for tumor cells rather than untransformed normal cells, which have a higher tolerance to BA [7,8]. However, the use of BA in tumor therapy has been limited due to its poor solubility and bioavailability. The development of derivatives like 3-acetyl-28-sulfamoyloxybetulin (CAI3) offers a potential approach to advance medical applications. CAI3 shows a higher cytotoxic activity and induces radiosensitivity in breast cancer cell lines [9,10]. Several studies indicate that BA induces intrinsic apoptotic pathways independently of upstream signaling and mutations that could lead to drug or chemotherapy resistance [5,11]. Nevertheless, the precise mechanisms of action of BA, especially in combination with radiation or chemotherapy, remain largely unexplored. Previous studies show that BA induces the expression of stress-induced gene *SESN2* in breast cancer cell lines and that treatment with BA and derivative CAI3 leads to an increased expression of genes involved in zinc homeostasis in breast cancer cells, including an overexpression of metallothionein 1 (*MT1*), zinc transporter 1 (*SLC30A1*), and S100 calcium and zinc binding protein (*S100P*) [12]. However, the role of zinc during treatment with BA or CAI3 remains largely unknown.

Zinc is one of the most crucial trace elements in cellular metabolism, necessary for numerous biochemical reactions. Its homeostasis is regulated through complex mechanisms involving zinc importers (ZIPs), zinc transporters (ZnTs), and metallothioneins (MTs) [13]. Zinc is a component of over 3000 proteins, influencing a wide range of physiological processes, including DNA and RNA synthesis, cell growth, and energy metabolism [14,15]. Reduced zinc levels have been associated with the early development of certain cancers, such as prostate, liver, and pancreatic cancer [16,17]. Notably, in some specific tumors, such as breast cancer, increased zinc levels could also be observed in malignant tissues [18]. Nevertheless, zinc deficiency is known to induce oxidative stress, resulting in DNA, protein, and lipid damage and significantly increasing the risk of cancer [19,20]. The significance of zinc as a tumor suppressor has been shown in various cancer types, as zinc concentrations that are tolerated in normal cells become cytotoxic in malignant cells, forming the basis for the development of zinc-associated chemotherapeutics [21]. In addition, zinc supplementation has shown promise in reducing side effects associated with chemotherapy [22,23]. The impact of the treatment with zinc in radiotherapy is also gaining attention, with initial studies showing increased radiosensitivity and apoptosis in different carcinomas [24,25].

In this study, it is investigated if a treatment of breast cancer cell line MDA-MB-231 with BA or CAI3 might possibly lead to an increase in intracellular zinc concentration. To this end, the significance of zinc supplementation alone or in combination with BA or CAI3 is investigated in breast cancer cell lines, focusing on cell viability, clonogenic survival, cell death rate, generation of reactive oxygen species, and radiosensitivity.

## 2. Materials and Methods

### 2.1. Cell Culture Conditions, Treatment of Cells

Human breast cancer cell lines MDA-MB-231, HS578T, T47D (kindly provided by Jürgen Dittmer, Department of Gynecology, Martin Luther University Halle-Wittenberg, Halle, Germany), and MCF-7 (Cell Lines Service GmbH, Eppelheim, Germany) were cultured at 37 °C and 5% CO_2_, with RPMI 1640 medium (Thermo Fisher Scientific, Waltham, MA, USA) containing 10% fetal bovine serum (Capricorn Scientific, Ebsdorfergrund, Germany), 1% sodium pyruvate (Gibco, Thermo Fisher Scientific), and 2% penicillin/streptomycin (Sigma-Aldrich, St. Louis, MO, USA). Cell line authentication was achieved by short tandem repeat (STR) DNA profiling to detect possible crosscontamination between cell lines. The cells were regularly tested for mycoplasma contamination by PCR. The cells were seeded in cell culture flasks (Greiner Bio-One, Kremsmünster, Austria) 24 h before treatment.

The chemical drugs betulinic acid (BA) and 3-Acetyl-28-sulfamoyloxybetulin (CAI3) (kindly provided by BioSolutions, Halle, Germany) were dissolved in dimethyl sulfoxide (DMSO; Sigma, Steinheim, Germany) to achieve a 20 mM stock solution. The purity of the substances was specified by the manufacturers as >99%. Zinc sulfate (ZnSO_4_; Sigma) was dissolved in water (sterile filtered) to achieve a 200 mM stock solution. The cells were treated with 20–40 µM BA, 20–40 µMCAI3, and/or 0–500 µM ZnSO_4_ for 24 h at 37 °C, depending on the assay performed.

### 2.2. Analysis of RNA Expression

Previous microarray analyses [12] show that genes such as *MT1E*, *MT1F*, *SLC30A1*, and *S100P* involved in the regulation of zinc homeostasis are differentially expressed by a treatment of breast cancer cells with BA or CAI3. RNA isolation, cDNA synthesis, and qRT-PCR were performed as previously described [12]. The TaqMan primers used are listed in Table 1. A no-template reaction was used as a negative control. Housekeeping gene *MMGT1* (membrane magnesium transporter 1) was used for normalization. The primers used for qRT-PCR were ordered from Sigma–Aldrich.

To quantify all qRT-PCR data, the delta–delta Ct method (∆∆Ct) was applied [26]. ∆Ct values were calculated as the variation between the Ct values of the gene of interest and the Ct values of the reference gene *MMGT1*. The average Ct value from cells treated with DMSO was chosen as the reference sample. The ∆∆Ct value was defined as the difference between the ∆Ct value of the treated sample and the ∆Ct value of the reference sample. The 2^−∆∆Ct^ represents the fold change in the mRNA expression level of the treated sample compared to the averaged reference sample.

### 2.3. FluoZin-3 Staining and Measurement of Intracellular Zinc

The quantification of intracellular free zinc was carried out using the zinc-specific fluorescent dye FluoZin-3-AM (Thermo Fisher Scientific). FluoZin-3-AM was always freshly dissolved in DMSO as a 10 µM stock solution, TPEN (Sigma) was dissolved in DMSO as a 2 mM stock solution, and pyrithione (Sigma) was dissolved in bidistilled water as a 200 mM stock solution.

MDA-MB-231 and HS578T cells were seeded in 6-well plates (Greiner Bio-One). After 24 h, the cells were treated with different concentrations of BA (20 µM and 40 µM), CAI3 (20 µM and 30 µM), and/or ZnSO_4_ (150 µM and 200 µM) for 24 h. Afterwards, the cells were incubated with trypsin-EDTA (Biochrom, Berlin, Germany) for 5 min at 37 °C. Trypsin was decanted, the cells were detached with culture medium, and the cell suspension was centrifuged at 800 rpm for 4 min in 15 mL tubes (Greiner Bio-One). Subsequently, the culture medium was decanted and the cells were stained by incubating with 1 µM FluoZin-3-AM in measurement buffer (5 mM glucose, 1 mM MgCl_2_, 1 mM NaH_2_PO_4_, 1.3 mM CaCl_2_, 25 mM HEPES, 120 mM NaCl, 5.4 mM KCl) for 30 min at 37 °C. The cells were washed by adding measurement buffer, and centrifugation at 800 rpm for 4 min. The cells were resuspended in 1 mL measurement buffer and the cell suspension was fractionated at 250 µL each in three wells of a 96-well plate (Greiner Bio-One): (1) cell sample (≙ F); (2) cell sample with 100 µM TPEN (≙ F_min_); (3) cell sample with 50 µM pyrithione and 100 µM ZnSO_4_ (≙ F_max_). Again, the samples were incubated for 10 min at 37 °C and resuspended in the wells.

The stained cells were analyzed for fluorescent signal with an LSR II Fortessa flow cytometer (BD Biosciences, Heidelberg, Germany). The measured mean fluorescence intensities of the three sample fractions (F, F_min_, and F_max_) described above were combined with the binding coefficient of FluoZin-3 (KD = 15 nM), and the concentration of free intracellular zinc ions (Zn^2+^) was determined according to Equation (1):

(1) $$\left[Zn^{2+}\right]=K_{D}*\left(\frac{F-F_{\min}}{F_{\max}-F}\right)$$

The average fluorescence intensities of 10,000 cells were used for evaluation.

### 2.4. Cytotoxicity

To determine the influence of intracellular zinc on the cytotoxicity of BA or CAI3, the CellTiter-Glo^®^ Luminescent Cell Viability Assay (Promega, Mannheim, Germany) was used. Breast cancer cells were seeded at 6000 cells/well in 96-well plates (Berthold Technologies GmbH & Co.KG, Bad Wildbad, Germany); after 24 h the growth medium was changed and the cells were treated with different concentrations of BA or CAI3 (20–40 µM) and/or ZnSO_4_ (0–500 µM) for another 24 h. Luminescence was measured using a Spark^®^ Multimode Microplate Reader (Tecan Group AG, Männedorf, Switzerland). IC50 values (half-maximal inhibitory concentration) were determined from the dose response curve calculated using Origin2019 (OriginLabCorp., Northampton, MA, USA).

### 2.5. Clonogenic Survival and Radiosensitivity

To estimate the effects of BA (20 µM and 40 µM), CAI3 (20 µM and 30 µM), and/or ZnSO_4_ (150 µM and 200 µM) on clonogenic survival, 24 h after treating the cells, a colony-forming assay was performed. Therefore, cells were trypsinized and a defined cell number between 150 and 20,000 (depending on cell line, treatment, and irradiation) were plated in 50 mL cell culture flasks. The cells were cultured at 37 °C and 5% CO_2_ and allowed to form colonies over 10 (HS578T) or 12 (MDA-MB-231) days. The colonies were fixed with formaldehyde (Roth, Karlsruhe, Germany) and stained with 10% Giemsa solution (Sigma). A minimal size of approximately 50 cells was set to be counted as a colony. Colony counting was made using the GelCount system (Oxford Optronics, Abingdon, UK).

Irradiation was performed to determine radiosensitivity 24 h after treatment with a Synergy linear accelerator (Elekta, Stockholm, Sweden). The cells were irradiated at different doses (0–10 Gy) depending on the cell line. Survival fraction (SF) and dose-modifying factor (DMF10) were defined as described previously [27]. Cell survival curves were fitted to a linear quadratic model (−lnS = αD + βD^2^) using Origin 2019 (OriginLab, Northampton, MA, USA).

### 2.6. Annexin V-PI Assay

For the evaluation of apoptosis using the Annexin V-PI method, MDA-MB-231 and HS578T cells were seeded in 6-well plates, and after 24 h, the cells were treated with BA (20 µM and 40 µM), CAI3 (20 µM and 30 µM), and/or ZnSO_4_ (150 µM and 200 µM) for another 24 h. The cells were then stained and analyzed by FACS using an LSR II Fortessa flow cytometer as previously described [27]. Upon assessment, annexin V- and PI-positive cells were classified as late apoptotic, annexin V-positive and PI-negative cells as early apoptotic, annexin V-negative and PI-positive cells as necrotic, and cells negative for both markers as vital.

### 2.7. Analysis of ROS

The formation of intracellular reactive oxygen species (ROS) was analyzed using the CM-H_2_DCF-assay, as previously described [28]. For this, the cells were cultured in RPMI 1640 medium without Phenol Red (Thermo Fisher Scientific) at least one week before testing and then seeded in 6-well plates (150,000 cells/well) for 24 h. After treatment with BA (20 µM and 40 µM)/CAI3 (20 µM and 30 µM) and/or ZnSO_4_ (150 µM and 200 µM) for 24 h, the cells were incubated with 0.5 µM ROS indicator CM-H_2_DCFDA (Thermo Fisher Scientific) in PBS (Sigma; complemented with CaCl_2_ and MgCl_2_) for 30 min at 37 °C according to the manufacturer’s suggestions. The cells were washed, trypsinized for 3 min, and resuspended in PBS. The average fluorescence intensities of 5000–10,000 (depending on the number of vital cells) detached cells were used for analysis with an LSR II Fortessa flow cytometer. The cells stained with CM-H_2_DCFDA were excited at 488 nm, and the fluorescent signal was measured using a 530/30 nm filter.

### 2.8. Statistical Analysis

All mean values and standard deviations (±SD) were obtained from at least three independent experiments. Statistical analyses were performed using Student’s *t*-test in Excel 16.0 (Microsoft Corporation, Redmond, WA, USA). Significant differences were based on *p*-values of ˂0.05, ≤0.01, or ≤0.001 in comparison with DMSO-treated cells and highlighted with asterisks (* *p* ≤ 0.05, ** *p* ≤ 0.01, *** *p* ≤ 0.001).

## 3. Results

### 3.1. Quantitative PCR Analysis

**Figure 1:**
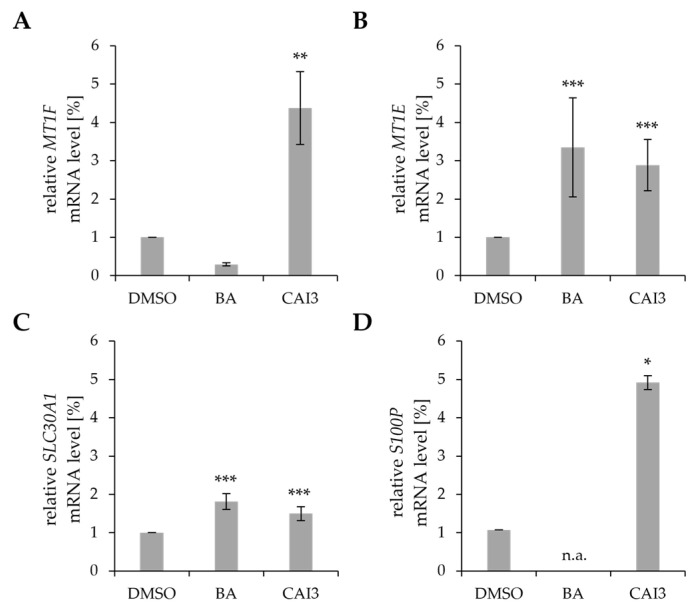
Relative mRNA expression genes involved in zinc homeostasis in MDA-MB-231 cells. Breast cancer cells were treated with BA or CAI3 for 48 h. Relative mRNA expressions of (**A**) *MT1F*, (**B**) *MT1E*, (**C**) *SLC30A1,* and (**D**) *S100P* were measured by qRT-PCR. Data represent mean values (±SD) of at least three independent experiments. All data were referred to DMSO-treated cells, and significant results are highlighted with asterisks (n.a., not analyzed; * *p* ≤ 0.05, ** *p* ≤ 0.01, *** *p* ≤ 0.001).

Four human breast cancer cell lines (MDA-MB-231, MCF-7, HS578T, and T47D) were subjected to a 48 h treatment with BA or CAI3 to uncover the genes and pathways potentially associated with the mechanism of action of BA or CAI3. Genes involved in zinc homeostasis *(SLC30A1*, *S100P*, *MT1F*, and *MT1E*) were particularly noticeable. PCR analyses showed that the mRNA expression levels of metallothioneins *MT1F* and *MT1E*, zinc transporter *SLC30A1*, and zinc binding protein *S100P* were significantly enhanced after treatment with BA or CAI3 (Table 2), with the exception of *MT1F* upon treatment with BA in MDA-MB-231 cells (Table 2, Figure 1).

### 3.2. Quantification of Intracellular Free Zinc

**Figure 2:**
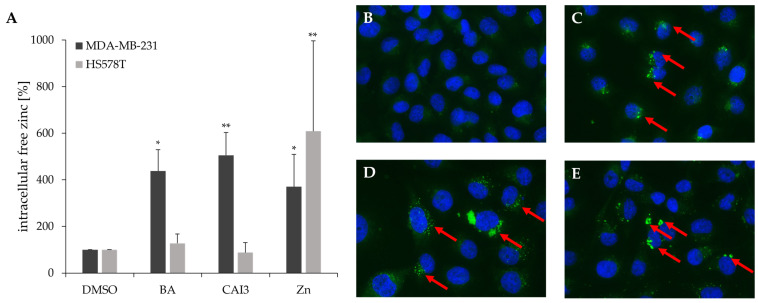
Intracellular free zinc concentration in breast cancer cells. MDA-MB-231 and HS578T cells were treated with BA, CAI3, or ZnSO_4_ for 24 h. After staining with 1 µM FluoZin-3-AM, cells were analyzed for intracellular free zinc by flow cytometry. (**A**) Data represent the mean values (+SD) of at least three independent experiments. All data were referred to DMSO-treated cells, and significant results are highlighted with asterisks (* *p* ≤ 0.05, ** *p* ≤ 0.01). Representative immunofluorescence staining of Zn^2+^ ions (green: FluoZin-3) and cell nuclei (blue: DAPI) in (**B**) untreated cells, (**C**) zinc, (**D**) BA, and (**E**) CAI3-treated cells in 20× magnification. The red arrows mark the accumulation of zinc in treated cells.

Previous studies showed that treatment with BA or CAI3 leads to dysregulation of zinc homeostasis genes in breast cancer cells. Consequently, the level of intracellular free zinc was investigated in breast cancer cell lines MDA-MB-231 and HS578T after treatment with BA, CAI3, and zinc. In MDA-MB-231 cells, treatment with BA caused a 4.4-fold (*p* = 0.04), CAI3 a 5.1-fold (*p* = 0.01), and zinc a 3.7-fold (*p* = 0.03) increase in intracellular free zinc concentration (Figure 2). However, in HS578T cells, treatment with BA and CAI3 had no significant effect on the intracellular free zinc concentration. A treatment with zinc caused a 6.1-fold (*p* = 0.01) increase (Figure 2). A combined treatment with BA or CAI3 and zinc showed no significant additional impact on intracellular free zinc concentration in both of the two breast cancer cell lines.

### 3.3. Cytotoxicity of BA or CAI3 and Zinc

**Figure 3:**
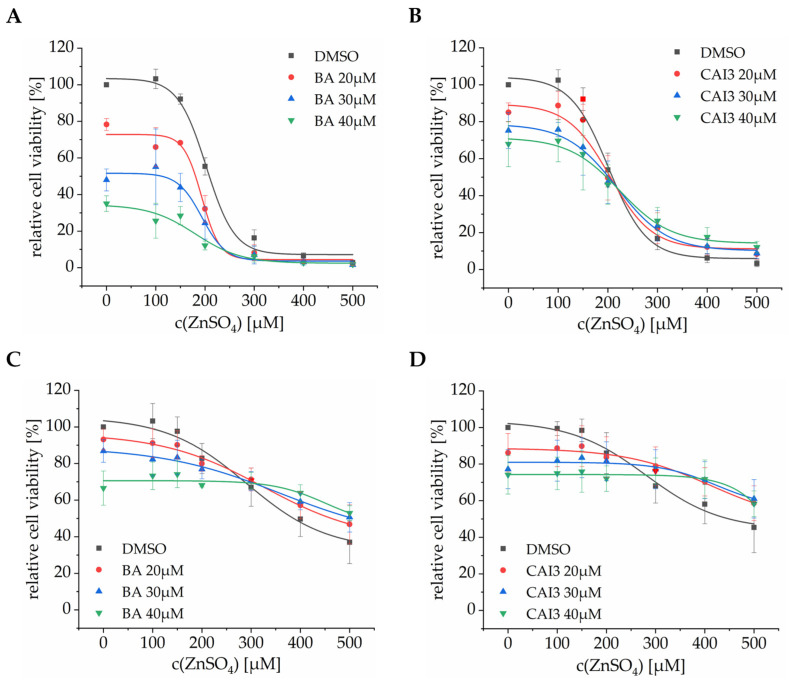
Dose-dependent effects on cell viability of zinc and BA or CAI3 as combined treatment in breast cancer cells. Cell viability was measured using a Cell Titer Glo^®^ assay. Cell lines (**A**,**B**) MDA-MB-231 and (**C**,**D**) HS578T were treated with ZnSO_4_ (100–500 µM) and (**A**,**C**) BA (20 µM, 30 µM, and 40 µM) or (**B**,**D**) CAI3 (20 µM, 30 µM, and 40 µM) for 24 h. Data represent mean values (±SD) of at least three independent experiments and were referred to DMSO-treated cells.

To investigate the effect of a treatment with zinc on the drug-induced cytotoxicity, a Cell Titer Glo^®^ assay (Promega, Mannheim, Germany) was performed with breast cancer cell lines MDA-MB-231 and HS578T, showing that cell viability decreased with increasing zinc and drug concentrations in both cell lines. During the co-treatment with zinc, the cytotoxicity of BA and CAI3 was influenced by the zinc concentration. Up to a zinc concentration of 150 µM (MDA-MB-231) or 200 µM (HS578T), a treatment with zinc had hardly any effect on the efficacy of the drugs. Treatment with 200 µM (MDA-MB-231) or 300 µM (HS578T) zinc showed the same cell viability regardless of drug concentration (except when treated with BA in MDA-MB-231 cells). The use of even higher zinc concentrations in combination with treatment with BA or CAI3 even showed slight cytoprotective effects (Figure 3B–D).

### 3.4. Effects of a Combined Treatment with BA or CAI3 and ZnSO_4_ on Clonogenic Cell Survival

**Figure 4:**
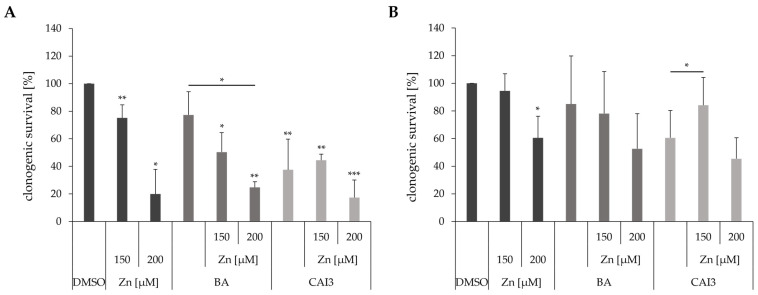
Clonogenic survival of breast cancer cells. (**A**) MDA-MB-231 and (**B**) HS578T breast cancer cells were treated with 150 µM and 200 µM ZnSO_4_ as single treatments and in combination with BA or CAI3 for 24 h. Data represent mean values (+SD) of at least three independent experiments. All data were referred to DMSO-treated cells, and significant results are highlighted with asterisks (* *p* ≤ 0.05, ** *p* ≤ 0.01, *** *p* ≤ 0.001).

A clonogenic survival assay was performed with MDA-MB-231 and HS578T breast cancer cells to investigate the effect of zinc supplementation on the clonogenic survival after treatment with BA or CAI3 (Figure 4). Based on pilot studies, zinc concentrations of 150 µM and 200 µM were selected to have a reliable effect on drug efficacy. Treatment with 150 µM zinc had only slight effects on clonogenic survival in both cell lines. However, after incubation with 200 µM zinc, a reduction of clonogenic survival was observed in MDA-MB-231 cells up to 19.9% (*p* = 0.02) and in HS578T cells up to 60.5% (*p* = 0.04). Simultaneous incubation of 150 µM zinc with the respective drug caused no significant effects on clonogenic survival compared to single treatment in both cell lines. Except in HS578T cells, treatment with 150 µM zinc even showed a cytoprotective effect on clonogenic survival after treatment with CAI3 with an increase in clonogenic survival of 23.6% (*p* ≤ 0.05) compared to that after treatment with CAI3 alone. In MDA-MB-231 cells, additional treatment with 200 µM zinc caused further reduction in clonogenic survival of 52.5% (BA; *p* = 0.02) and 20.1% (CAI3), respectively, compared to the respective treatment without zinc. In HS578T cells, the same treatment caused further reductions in clonogenic survival of 32.4% (BA) and 15.1% (CAI3), respectively (Figure 4).

### 3.5. Effects of a Combined Treatment with BA or CAI3 and ZnSO_4_ on Cell Death

**Figure 5:**
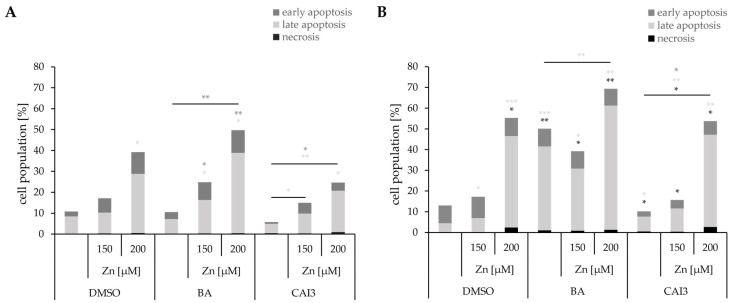
Evaluation of necrosis and apoptosis using the Annexin V-PI method in breast cancer cells. Cell lines (**A**) MDA-MB-231 and (**B**) HS578T were treated with 150 µM and 200 µM ZnSO_4_ and BA or CAI3, respectively, for 24 h. Data represent mean values of at least three independent experiments. All data were referred to DMSO-treated cells and significant results are highlighted with asterisks (* *p* ≤ 0.05, ** *p* ≤ 0.01, *** *p* ≤ 0.001).

Quantification of necrotic (Q1), late apoptotic (Q2), and early apoptotic (Q4) cells was performed using Annexin V-PI analysis (Figure 5). Treatment with 150 µM zinc had only slight effects on cell death rate, whereas 200 µM zinc resulted in a strong induction of cell death in both cell lines (MDA-MB-231: 39.3% (0.5% necrotic; 28.3% late apoptotic; and 10.5% early apoptotic cells); HS578T: 55.3% (2.4% necrotic; 44.2% late apoptotic; and 8.7% early apoptotic cells). Simultaneous incubation of 150 µM zinc with each drug caused a low increase in cell death rate compared to single drug treatment in both cell lines. Except in HS578T, treatment with 150 µM zinc showed a cytoprotective effect on cell death after treatment with BA with a reduction of late apoptosis of 10% compared to that after treatment with BA alone. Additional treatment with 200 µM zinc had no significant effect on necrosis but significantly increased late and early apoptosis by 38.9% in MDA-MB-231 and by 19% in HS578T compared to that after treatment with BA alone (Figure 5). The combined treatment with 200 µM zinc and CAI3 resulted in an increase in necrosis as well as late and early apoptosis summing up of 19% (MDA-MB-231) and 43.4% (HS578T) compared to that after a treatment with CAI3 alone (Figure 5).

### 3.6. Effects of a Combined Treatment with BA or CAI3 and ZnSO_4_ on Reactive Oxygen Species (ROS) Formation

**Figure 6:**
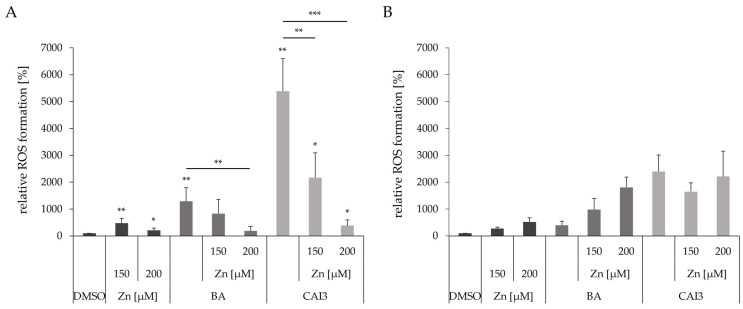
ROS formation in breast cancer cells using the CM-H_2_DCF-assay. Cell lines (**A**) MDA-MB-231 and (**B**) HS578T were treated with 150 µM and 200 µM ZnSO_4_ and BA or CAI3, respectively, for 24 h. Data represent mean values (+SD) of at least three independent experiments and were referred to DMSO-treated cells. Significant results are highlighted with asterisks (* *p* ≤ 0.05, ** *p* ≤ 0.01, *** *p* ≤ 0.001).

ROS formation was investigated after treating cells with zinc and BA or CAI3 using the CM-H_2_DCF-assay (Figure 6). A treatment with 150 µM zinc led to a 4.7-fold (*p* = 0.005) and 2.8-fold increase in ROS production in MDA-MB-231 and HS578T (*p* = 0.01) breast cancer cells, respectively. While treatment with 200 µM zinc in MDA-MB-231 cells showed only a slight 2-fold (*p* = 0.04) increase, the ROS levels in HS578T cells continued to rise a 5.6-fold increase (*p* = 0.02). A combined treatment with zinc and BA or CAI3 caused an inhibition of a BA-induced or a CAI3-induced increase in ROS production in MDA-MB-231 cells: the incubation with 200 µM zinc resulted in a significant decrease in ROS production of 7-fold after BA (*p* = 0.002) and of 14-fold after CAI3 (*p* = 0.0002) treatment compared to their respective individual substance treatments. However, in HS578T cells, an additional treatment with 200 µM zinc led to a significant 4.6-fold (*p* = 0.01) increase in ROS production compared to that after treatment with BA alone (4-fold, *p* = 0.04), whereas it showed no significant effect on ROS production compared to that after treatment with CAI3 alone (26-fold, *p* = 0.02) (Figure 6).

### 3.7. Effects of a Combined Treatment with BA or CAI3 and ZnSO_4_ on Radiosensitivity

**Figure 7:**
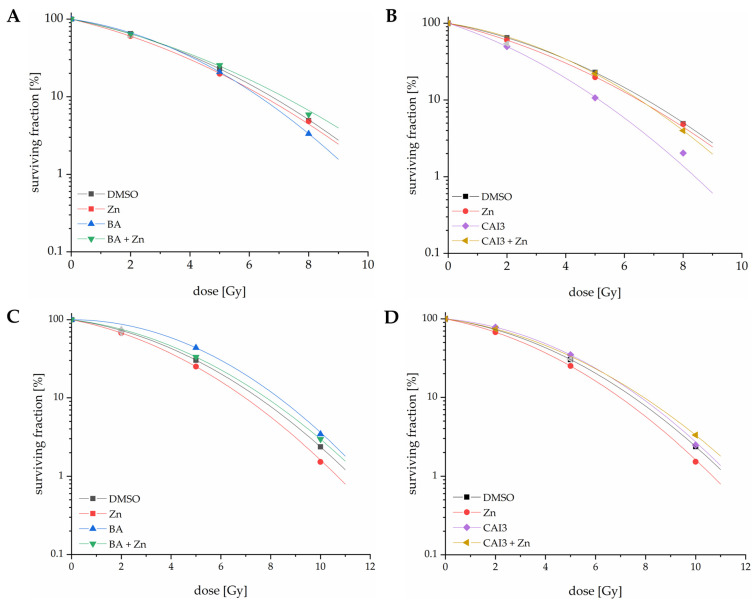
The influence of zinc on radiosensitivity of breast cancer cells. Cell lines (**A**,**B**) MDA-MB-231 and (**C**,**D**) HS578T were treated with 150 µM ZnSO_4_ and (**A**,**C**) BA or (**B**,**D**) CAI3. After a 24 h treatment, cells were irradiated with different doses depending on the cell line. Afterwards, 150 to 20,000 single cells were plated depending on the cell line and radiation dose. After 10 (HS578T) or 12 (MDA-MB-231) days of colony formation, the colonies were stained and counted. Data represent mean values of at least three independent experiments and were referred to non-irradiated cells for each treatment.

The radiosensitivity of combined treatment with zinc and BA or CAI3 was observed after irradiation with 2 Gy, 5 Gy, and 8 Gy (MDA-MB-231) or 10 Gy (HS578T). Treatment with BA had almost no effect on radiosensitization (DMF10 = 1.05), whereas treatment with CAI3-radiosensitized MDA-MB-231 cells (DMF10 = 1.29, *p* = 0.09) consistent to [10]. In addition, a treatment with zinc showed no significant effects on the radiosensitivity of MDA-MB-231 breast cancer cells (Figure 7A,B). However, the combined treatment with zinc and BA or CAI3 had radioprotective effects. In particular, the combined treatment with zinc and CAI3 resulted in a significant reduction of the DMF10 value to 1.04 (*p* = 0.008) compared to the treatment with CAI3 alone (DMF10 = 1.29) (Figure 7B).

In contrast, radiosensitizing effects could be observed in HS578T cells after a treatment with zinc (DMF10 = 1.11, *p* = 0.03), whereas a treatment with BA or CAI3 did not affect the radiosensitivity of HS578T breast cancer cells. The additional treatment with zinc had no effect on the radiosensitivity of the HS578T cells treated with BA or CAI3 (Figure 7C,D).

## 4. Discussion

BA, a natural compound derived from plane tree bark, exhibits a variety of antitumor effects. Examination of the transcriptional profiles regarding the cytotoxicity of BA in 60 NCI cell lines indicated the involvement in different mechanisms [29]. BA and derivatives such as CAI3 exhibit selective cytotoxic effects, induce apoptosis, inhibit migration, and show radiosensitizing effects in tumor cells [10,30,31,32,33]. Previous microarray analyses showed that a treatment with BA or the derivative CAI3 disrupted the regulation of genes involved in zinc homeostasis in breast cancer cells, including upregulation of metallothionein 1 (*MT1*), zinc transporter 1 (*SLC30A1*), and S100 calcium-binding protein (*S100P*).

Compared to healthy controls, decreased serum zinc levels have been observed in breast cancer patients [34]. However, elevated zinc levels were observed in tissues of breast cancer patients compared to those of a healthy control group [35]. An increased intracellular uptake of zinc and enzymatic activity in highly proliferative tumor cells are responsible for an increased influx of zinc and result in a decreased serum level and an overload of intracellular zinc level. Metastatic breast cancer cells have an increased zinc content compared to that in normal breast cancer cells, which is essential for the modulation of the microenvironment [36]. Additionally, Vogel–Gonzalez indicated that zinc promoted the tumorigenic behavior of breast cancer cells. A therapeutic approach with zinc oxide nanoparticles (ZnO-NP) showed a selective inhibition of tumor cells, an induction of apoptosis and necrosis, an inhibition of migration and attachment, and an induction of ROS production in breast cancer cells [37,38,39]. In addition, the in vivo inhibitory effect of zinc supplementation in the form of ZnO-NPs on breast cancer growth in rats confirmed the relevance of zinc for cancer metabolism and the potential of zinc as an attractive chemotherapeutic approach for breast cancer [40]. In this context, the aim of the present study was to investigate the significance of zinc supplementation during treatment with BA or CAI3 combined with radiotherapy in breast cancer cell lines MDA-MB-231 and HS578T.

Previous microarray analyses of breast cancer cells treated with BA or CAI3 showed dysregulation of several genes involved in zinc homeostasis. In further investigations, four breast cancer cell lines were screened for mRNA expression of *MT1F*, *MT1E*, *SLC30A1*, and *S100P* after treatment with BA or CAI3 and showed an upregulation of *MT1F*, *MT1E*, *SLC30A1*, and *S100P* in four breast cancer cell lines (Table 2). These results indicate that zinc homeostasis is disturbed, which is confirmed by measurement of intracellular free zinc level: In MDA-MB-231 cells, a strong increase in intracellular free zinc level was observed after treatment with BA, CAI3, and zinc (Figure 2). However, only a marginal effect on intracellular free zinc level was observed in HS578T cells. This may depend on the cell line-specific effects or concentration of BA, CAI3, and zinc, respectively. However, defense mechanisms of HS578T cells against increasing zinc concentrations are also conceivable. They might compensate for the dysregulation of zinc homeostasis in HS578T cells better than MDA-MB-231 by the regulation of genes/proteins relevant for the intracellular zinc level, such as ZnTs, ZIPs, or MTs. Chandler et al. observed that different breast cancer cell lines show specific mRNA and protein expression profiles for members of ZnT or ZIP families, which resulted in differences in intracellular zinc accumulation [41].

Nevertheless, both of the examined breast cancer cell lines showed an increasing cytotoxicity after treatment with increasing zinc concentrations (100–500 µM). However, no significant additive effects were observed in combination with a treatment with BA or CAI3. In addition, there were even protective effects, and the cells appeared to be more resistant to chemotherapeutic treatment at high zinc concentrations (Figure 3). Additionally, both cell lines showed anticancer effects after a treatment with zinc concerning clonogenic survival and apoptosis, as well as enhancing effects on inhibition of clonogenic survival and the induction of cell death after treatment with BA or CAI3 (Figure 4 and Figure 5). Other studies also show a reduction in viability in breast cancer cells using zinc pyrithione or Zn-NPs as well as inhibition of cell proliferation, clonogenicity, migration, invasion, and induction of apoptosis, autophagy, and cuproptosis [37,38,39,42]. Even in more complex models, a smaller number and slower growth of spheroids could be observed, as well as a decrease in the number and length of vessels in the CAM (chorioallantoic membrane) model and even slower tumor growth in vivo in the mouse model [37,38,42,43].

At present, there are only a few studies in which the combined effect of chemotherapy and zinc was investigated in breast cancer cells. Hu et al. showed that the use of ZnO-NPs sensitized normal MCF-7 cells as well as drug-resistant MCF-7/ADR breast cancer cells to treatment with doxorubicin. They postulated that ZnO-NPs induced autophagy, resulting in cell death through ROS generation [38]. Quercetin-loaded ZnO-NPs had a higher potential to induce apoptosis, oxidative stress, and mitochondrial damage in breast cancer cells and caused a greater reduction in tumor growth and tumor-associated toxicity in the liver and kidney in a mammary adenocarcinoma model compared to those after a single chemotherapeutic treatment [43]. Similar results were obtained in prostate and ovarian cancer cells, where zinc/ZnO-NPs mediated increased chemosensitivity (paclitaxel, doxorubicin), which was reflected in reduced viability, clonogenic survival, invasion, migration, and increased apoptosis [44,45,46].

BA and its derivatives are known to decrease the mitochondrial membrane potential, which results in mitochondrial dysfunction and the generation of ROS. In this study, the enhanced ROS generation after treatment with BA or CAI3 could be confirmed. However, zinc is described to have pro-oxidant and antioxidant effects [47], which are apparently dependent on the zinc concentration and cell line (Figure 6). Stephankova et al. supported this phenomenon by describing the opposite effects of ZnO-NPs on ROS generation in two different triple-negative breast cancer cells [39]. Additionally, the two studied breast cancer cell lines showed different responses to the formation of ROS after incubation with BA or CAI3 in combination with zinc supplementation. In MDA-MB-231 cells, the addition of zinc to treatment with BA or CAI3 had a strong antioxidative effect (Figure 6). There is evidence that zinc can induce antioxidant molecules/enzymes like glutathione, SOD (superoxide dismutase), and Nrf2 (nuclear factor erythroid 2-related factor 2) [47]. In contrast, in HS578T cells, the supplementation of zinc in combination with a treatment with BA or CAI3 leads to pro-oxidative effects (Figure 6). In this case, the inhibition of mitochondrial function by zinc due to destruction of the mitochondrial membrane potential further increases ROS production, which enhances oxidative stress [47].

The contrasting function of zinc as a pro- and antioxidant in the two studied breast cancer cell lines is also reflected in the effects of irradiation after treatment with BA or CAI3 with and without zinc (Figure 7). In MDA-MB-231 cells, where zinc acts as an antioxidant, zinc supplementation to treatment with BA or CAI3 overcomes the radiosensitizing effects of BA and CAI3. In HS578T cells, where the addition of zinc had only a small effect on the ROS formation, treatment with zinc had a radiosensitizing effect but no significant effect on radiosensitivity in the case of a combined treatment with BA or CAI3. To date, there have been only a few studies investigating the role of zinc in the radiosensitivity of tumor cells. Radiochemotherapy consisting of a chemotherapeutic agent and zinc showed radiosensitizing effects in cervical and nasopharyngeal cancer cells compared to controls [24,25]. For breast cancer cells, Arab-Bafrani et al. also described the radiosensitizing effects of ZnO bio-nanocomposites loaded with chemotherapeutic agents, associated with increased ROS generation and apoptosis rate due to increased radiation-induced complex DNA breakage as well as weakened DNA repair mechanisms [48]. In addition, chemotherapy with zinc and clioquinol caused enhanced DNA double-strand breaks and apoptosis in MCF-7 breast cancer cells, resulting in radiosensitizing effects [25]. However, Zn-MTs are able to protect against radiation-induced DNA damage better than other antioxidants such as GSH or other Cu-binding MTs [49].

In terms of clinical use, zinc is discussed as an adjunctive therapy, particularly with radiotherapy, as it may reduce the side effects on normal tissue [50]. In detail, administration of zinc delayed the beginning, shortened the duration and reduced the intensity of radiation-induced mucositis in head and neck cancer patients [51,52]. In addition, zinc could improve the impact of radiotherapy on oral pain, loss of taste, and dermatitis [52,53,54]. The findings of this study indicate that tumor cells could also benefit from zinc administration, suggesting to assess the benefit of a treatment with zinc during radiotherapy on an individual basis, but further studies are needed to investigate the clinical relevance of zinc and BA or BA derivatives for radiotherapy.

In conclusion, zinc seems to play an important role in breast cancer and could thus be considered as an individual therapy option for breast cancer patients.

## Figures and Tables

**Table 1:** TaqMan primer.

Gene	Assay ID	Product Length
*MMGT1*	Hs00953954_m1	100 bp
*MT1E*	Hs01652848_g1	146 bp
*MT1F*	Hs04398568_g1	166 bp
*SLC30A1*	Hs00253602_m1	97 bp
*S100P*	Hs00195584_m1	73 bp

SLC30A1, solute carrier family 30 member 1; MT1E, metallothionein 1E; MT1F, metallothionein 1F; S100P, S100 calcium binding protein P.

**Table 2:** Relative mRNA expression (fold change) caused by treatment with BA or CAI3 referred to DMSO-treated cells.

	MDA-MB-231		MCF-7		T47D		HS578T	
Gene	BA	CAI3	BA	CAI3	BA	CAI3	BA	CAI3
*MT1F*	0.3	4.4 **	2.9	2.3 *	14.3	7.8 **	n.d.	n.d.
*MT1E*	3.4 ***	2.9 ***	2.3	2.4	n.d.	n.d.	2.5	4.1 ***
*SLC30A1*	1.8 ***	1.5 ***	1.5 ***	1.7 ***	1.4	2.0 **	1.3 **	1.7 **
*S100P*	n.a.	4.4 *	n.a.	2.1 *	n.a.	1.7	n.a.	6.7

MT1F, metallothionein 1F; MT1E, metallothionein 1E; SLC30A1, solute carrier family 30 member 1; S100P, S100 calcium-binding protein; n.d., not detectable; n.a., not analyzed; * ≤0.05; ** ≤0.01; *** ≤0.001.

## Data Availability

The data that support the findings of this study are available from the corresponding author, AG, upon request.
